# Acceptability, appropriateness, willingness to use, and perceptions towards HIV self-testing among adolescent girls and young women in rural Northern Uganda: a baseline formative cross-sectional study

**DOI:** 10.1186/s43058-025-00822-w

**Published:** 2025-12-11

**Authors:** Ronald Olum, Morrish Obol Okello, Freddy Eric Kitutu, Elvin H. Geng, Philippa Musoke

**Affiliations:** 1 Department of Community Health and Behavioral Sciences, Makerere University School of Public Health, Kampala, Uganda; 2 Department of Epidemiology, Johns Hopkins Bloomberg School of Public Health, Baltimore, MD USA; 3 Department of Pharmacy, Makerere University School of Health Sciences, Kampala, Uganda; 4 Department of Women’s and Children’s Health, International Child Health and Migration, Uppsala University, 751 85 Uppsala, Sweden; 5 Division of Infectious Diseases, School of Medicine, Washington University in St Louis, St Louis, MO USA; 6 Makerere University-Johns Hopkins University Research Collaboration, Kampala, Uganda; 7 Department of Paediatrics and Child Health, College of Health Sciences, Makerere University, Kampala, Uganda

## Abstract

**Background:**

Adolescent girls and young women (AGYW) in Uganda are at higher risk for and bear a significant HIV burden, accounting for 25% of new infections. Despite improved HIV testing services, AGYW in rural areas face barriers to facility-based testing due to stigma, physical access barriers, and confidentiality concerns. This study assessed the acceptability, appropriateness, willingness to use, and perceptions of HIVST among AGYW in Northern Uganda.

**Methods:**

This cross-sectional study was part of a baseline assessment for a quasi-experimental trial evaluating community-led HIVST among AGYW aged 15–24 years in 5 sub-counties in Omoro District. Data were collected using systematic random sampling of households, with trained research assistants administering structured questionnaires on tablets. The survey captured demographic characteristics, sexual history, HIV knowledge, prior testing practices, and attitudes toward HIVST. Factors influencing willingness to use HIVST were analyzed using simple logistic regression in Stata 18.0.

**Results:**

Among 415 AGYW (median age 19 years, IQR 17–22), 23.1% had at least a secondary education, 41.4% were married or cohabiting, and 16.9% had been in more than one marriage or union. Sexual activity was reported by 74.2%, with a median age at first intercourse of 16 years (IQR 15–18); 12.7% reported having multiple sexual partners in the past year. Although 75.4% had been tested for HIV, only 28.0% had heard of HIVST, and 17.5% of these had used it. More than two-thirds of the participants found HIVST acceptable, appropriate, and feasible. Willingness to use HIVST was high (93.0%), with preferences for blood-based (53.3%) and oral fluid-based tests (46.3%). Willingness to use HIVST was associated with older age (COR 1.19, 95% CI 1.03–1.37, *p* = 0.017), ever having had sexual intercourse (COR 2.67, 95% CI 1.25–5.71, *p* = 0.011), and prior HIV testing (COR 2.32, 95% CI 1.07–5.04, *p* = 0.033). Preferred access points included government health facilities (64.8%), community hotspots (57.8%), friends (33.3%), and CHWs (21.9%). Over half (61.0%) desired additional support when testing, mainly from health workers (69.6%) and friends (26.1%). Anticipated challenges included interpretation results (57.1%), insufficient test usage information (53.7%), and performing the test correctly (52.3%).

**Conclusion:**

Our findings indicate high acceptability of HIVST among AGYW in rural northern Uganda, significantly higher in older individuals, prior sexual activity, and prior HIV testing experience. Targeted implementation strategies addressing knowledge gaps, providing beneficiary support, and leveraging existing community structures could further optimize HIVST uptake. Research on sustainable community-led models of HIVST distribution will be critical to reaching underserved AGYW, reducing undiagnosed HIV infections, and strengthening HIV prevention and care outcomes in this key population.

**Supplementary Information:**

The online version contains supplementary material available at 10.1186/s43058-025-00822-w.

Contributions to the literature

- This study is among the first to assess HIV self-testing (HIVST) implementation outcomes among adolescent girls and young women (AGYW) in rural Northern Uganda.
- We demonstrate high acceptability, appropriateness, and willingness to use HIVST in a conflict-affected setting with limited prior exposure to HIVST.
- Our findings highlight key factors influencing HIVST uptake, including age, sexual history, prior testing, and confidence in using/testing kits.
- We recommend context-sensitive, community-led HIVST distribution models to improve coverage among AGYW in underserved areas.
- The study informs future implementation strategies by identifying AGYW preferences and barriers, which are critical for designing equitable HIV prevention programs.

## Introduction

Substantial progress has been made in curbing the global HIV epidemic, with new infections declining by 39% worldwide and by 56% in sub-Saharan Africa (SSA) since 2010 [1]. Despite these successes, adolescent girls and young women (AGYW) aged 15–24 in SSA continue to be disproportionately affected by the epidemic [1]. In 2023 alone, an estimated 360,000 AGYW acquired HIV worldwide, 77.5% of whom lived in SSA [2], while Uganda recorded approximately 12,000 new HIV cases among AGYW that same year. A combination of biological, socio-economic, and cultural factors not only increases the risk of HIV transmission among AGYW but also hinders access to effective prevention and treatment services [3].

The 95–95–95 UNAIDS 2030 global targets are within reach [4], and recent data indicate that 86% of people living with HIV knew their status in 2023, 89% were on antiretroviral therapy, and 93% had a suppressed viral load [1]. Uganda has made significant progress with an estimated cascade of 92–90–94 in 2023 [1]. A crucial part of achieving these targets is increasing HIV testing uptake to enhance case identification and ensure timely linkage to care and support services [5]. Despite overall gains, only 35% of AGYW in SSA have ever been tested for HIV, and just 65% of those living with HIV know their status, leaving an estimated half a million undiagnosed [6].

HIV self-testing (HIVST) is a promising strategy to increase HIV testing uptake, especially among underserved populations like AGYW [7]. HIVST allows individuals to collect their samples, perform the test, and interpret the results privately, reducing stigma and increasing convenience, overcoming traditional barriers to facility-based testing [7, 8]. Two HIVST technologies exist, including blood-based and oral fluid-based tests [9, 10], each with various levels of preference and acceptability [10]. Studies indicate that HIVST can increase testing frequency, enhance users’ autonomy, and reduce the strain on already overburdened healthcare systems [11, 12].

HIVST is highly acceptable and feasible among AGYW in SSA [13–16]. Implementation studies across SSA have also demonstrated that HIVST can be successfully integrated into existing health systems, enhancing access and uptake of HIV testing services [17]. However, utilization of HIVST among young women in SSA remains sub-optimal, at only 2.17% [18]. In Uganda, research among female students at Makerere University revealed that while over 93% were willing to use HIVST, only 19% had ever done so, suggesting a significant gap in willingness and actual use [19]. Among young women residing in fishing communities in southwestern Uganda, 100% expressed willingness to use HIVST kits if they were made available [20]. Secondary distribution by peers particularly enhanced uptake and acceptability in Uganda [16, 21, 22].

Despite the robust evidence supporting HIVST in sub-Saharan Africa and other parts of Uganda, limited data exist on its implementation in Northern Uganda. This region, still grappling with the long-term socio-economic impacts of prolonged civil conflict, has unique challenges that may influence the uptake of HIVST among AGYW. The HIV prevalence among AGYW here is 9.7%, nearly double the national rate of 5.1% [23]. Therefore, the present study aimed to assess the acceptability, appropriateness, feasibility, and preferences of HIVST among AGYW in rural Northern Uganda. We then evaluate willingness to use HIVST in this vulnerable, underserved population using the health belief model and additional factors we deemed critical to decision-making among AGYW.

## Methods and materials

### Study design

This cross-sectional study was part of a larger quasi-experimental study evaluating the implementation of a peer-led HIV self-testing model facilitated by community health workers (CHWs) for AGYW in rural Northern Uganda. The study protocol is published elsewhere [24]. This manuscript reports quantitative findings from the baseline formative survey conducted between July and September 2024. The manuscript adheres to Strengthening the Reporting of Observational Studies in Epidemiology (STROBE) guidelines for cross-sectional studies (Supplementary File 1).

### Study setting

The current study was conducted in Omoro district, located south of Gulu and North of Kampala, Uganda’s capital city. Omoro is one of the districts affected by over two decades of civil conflict in Northern Uganda and suffered mass destruction of property, displacement, and loss of lives. It is among the least developed districts in the region and the country, with a GDP per capita of 183 USD [25]. Lalogi Health Center IV is the district’s largest public health facility, with 5 Health Center IIIs and 15 Health Center IIs. Omoro is divided into Tochi and Omoro counties and has twelve sub-counties.

### Selection criteria

All adolescent girls and young women aged 15–24 years who had lived in the selected parishes for at least three months and did not plan to leave their respective villages in the next year were eligible to participate in the study after providing informed consent. AGYW with a confirmed diagnosis of HIV/AIDS and/or on antiretroviral therapy prior to this study were excluded.

### Sample size and sampling techniques

The sample size was calculated using Epi Info StatCalc for population surveys and was powered with acceptability as the primary outcome variable. As of the 2014 national census, which was available at the time of this study, females made up 51% of the population in Omoro [26], leading to an estimated population of 16,383 AGYWs. At an average population growth rate of 3.2% between 2014 and 2022, the estimated population of AGYW in Omoro district was 21,753 by the end of 2023. To estimate the acceptability of HIVST among AGYW with an estimated population size of 21,753, expected acceptability of 50% since no previous studies have been conducted in this region, a margin of error of ± 5% at a 95% confidence level, and a design effect of 1.0, we determined that a sample size of at least 377 AGYW is required. To cater for non-response and loss-to-follow-up, an additional 10% of the sample size was added, leading to a final sample size of 415 AGYW.

A multistage random sampling method was employed to select study participants representative of the district’s population. Initially, we purposively selected five sub-counties from the twelve available in the district, based on a discussion with the district health team, which took into account the district’s HIV burden. The sample size was divided equally across the five sub-counties, giving 83 per sub-county. Then, one parish was selected randomly from each of the five sub-counties as a study site. We then randomly selected two villages from each parish to participate in the study. The study participants were selected through systematic random sampling, depending on the number of households obtained from the local council leaders and the desired sample size per sub-county.

Because reliable household data were unavailable, we used the adolescent girls and young women (AGYW) population as a proxy to calculate the sampling interval. The estimated AGYW population in Omoro District was 21,753, which, when divided across 168 villages, yielded an average of 129 AGYW per village. To obtain 42 participants per village, a sampling interval of three was applied. Within each village, a research assistant, guided by a community leader, determined a random starting point and direction. From that point, every third household was approached, and one eligible AGYW was recruited until the target sample size was achieved. If no eligible AGYW was found in the selected household, or if she declined participation, the next household was approached.

**Fig. 1:**
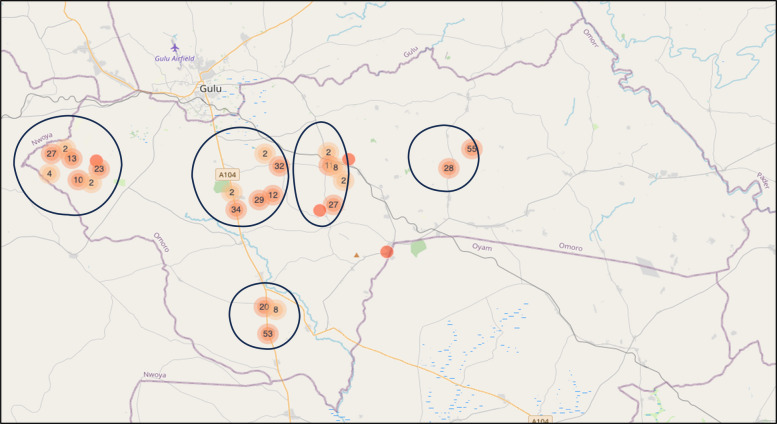
A map of Omoro District showing the locations where the participants were recruited. The circles represent the five sub-counties they were recruited from, namely, Bobi, Koro, Lakwana, Lalogi, and Ongako. This map was created in *KoboToolbox* and visualized with *Leaflet*, an open-source *JavaScript* library for interactive maps. The basemap tiles are provided by *OpenStreetMap* contributors under the Open Data Commons Open Database License (ODbL; see https://www.openstreetmap.org/copyright)

Figure 1 shows the distribution of the study participants by location.

### Study outcomes and measurements

#### Independent variables

We collected sociodemographic data (age, marital status, education, employment, income, and access to healthcare) and sexual and reproductive health history (prior sexual intercourse, number of sexual partners, previous pregnancies, and history of sexually transmitted diseases). Informed by the health belief model, we also collected data on their HIV risk perception, including Likert item questions on perceived risk, perceived severity, perceived barriers, cues to action, self-efficacy, and perceived benefits of HIV testing. The questionnaire also included questions on prior awareness, access, and utilization of HIV testing (including HIVST).

#### Dependent variables

The implementation outcomes of acceptability, appropriateness, and feasibility were defined according to the Proctor Framework [27]. Acceptability was defined as the AGYW's perception that HIVST is ‘agreeable, palatable, or satisfactory.’ Appropriateness was defined as the proportion of AGYW who perceived HIVST as a relevant and fitting solution for HIV testing challenges. Feasibility was defined as the extent to which HIVST ‘can be successfully used or carried out by AGYW within a given agency or setting.’ We adopted constructs from the Acceptability of Intervention Measure (AIM), the Intervention Appropriateness Measure (IAM), and the Feasibility of Intervention Measure (FIM) [28]. Since the original measurement tools were translated into Acholi, some constructs were not linguistically distinct in translation. Therefore, we adapted the items to ensure conceptual clarity and cultural relevance.

For acceptability (AIM), we retained two questions that could be clearly translated and meaningfully distinguished in Acholi: whether AGYW “liked” and “welcomed” HIVST. For appropriateness (IAM), all the original questions were translatable using the Acholi equivalent of “appropriate.” To enhance contextual relevance, we included one core item assessing general appropriateness (“HIVST seems appropriate”) and added four items that capture key appropriateness aspects in this setting: privacy, autonomy, accuracy, and stigma. For feasibility (FIM), all four original constructs had similar Acholi equivalents, and we retained the item assessing ease of use as a core measure. Additionally, we included two supplementary items to evaluate dimensions of feasibility that are particularly relevant in this context: the ability to perform the test and the ability to interpret the results. Finally, we assessed the willingness to use HIV self-testing with a single five-point Likert question: If made available, to what extent would you be willing to use the HIV self-testing kits?

### Study procedures

With support from a community guide, trained research assistants approached eligible households within the sampling frame, applying the sampling criteria to determine if there was an eligible participant in each household. They then sought permission from the household head (either the parents, guardians, or spouses) to recruit an eligible participant for the study. After obtaining written informed consent from the eligible participant, the research assistant then administered a structured questionnaire to each participant using KoBo Toolbox (Kobo, Cambridge, Massachusetts, USA) deployed on tablets. The interviews were conducted in Acholi and Langi, the most spoken dialects in the district.

### Data management and analysis

Data from the interviews were uploaded to secure cloud storage and exported to Stata 18.0 (StataCorp LLC, College Station, Texas, USA) for cleaning, coding, and analysis. At the univariate level, we described participants’ characteristics using frequencies and percentages for categorical variables, as well as medians and interquartile ranges for continuous variables, since they were not normally distributed. The implementation outcomes, measured on ordinal Likert scales, were analyzed descriptively, and the frequencies and percentages were presented as a heat map. Willingness to use HIVST, if made available, was collapsed into a binary outcome: “yes” for those who selected “very willing” and “somewhat willing, " and “no” for the remaining Likert scale responses. Factors associated with the willingness to perform HIVST were assessed using simple logistic regression models. The factors included in the model were selected based on the literature review guided by the health belief model. A *p*-value of < 0.05 was considered statistically significant. We could not perform a multivariable logistic regression due to the low proportion of participants unwilling to perform HIVST (7%, *n* = 29), as the small number of events would have led to unstable estimates, insufficient power, and a high risk of overfitting in the model.

## Results

### Characteristics of the study participants

A total of 415 AGYW were recruited (100% response rate), with a median age of 19 years (IQR: 17–22), and slightly over half (51.8%) were adolescents aged 15–19. While 6.5% had no formal education, 31.6% completed primary school, and 23.1% attained secondary education or higher. About half (50.4%) were single, 13.7% were married, and 27.7% were cohabiting. Among those who had ever been in a union (49.6%), the median age at first marriage was 17 (IQR: 16–19). Health decisions were most often made by parents (47.2%), followed by spouses/partners (24.6%), and oneself (11.1%). Agriculture was the primary income source (58.1%), though 30.4% had no source of income, and 36.6% earned under 50,000 UGX monthly. Over half (58.3%) lived 1–5 km from a health facility, and walking (84.6%) was the most common means of transport.

About 72.0% had ever had sexual intercourse, with a median age of 16 years at first intercourse. Of those sexually active in the past 12 months, 80.6% had one partner, while 19.4% had multiple partners. Only 3 (1.4%) self-reported being diagnosed with an STI: two with syphilis and one with gonorrhea. Up to 75.1% had ever been pregnant, with most having one (48.7%) or two (33.9%) prior pregnancies. Table 1 summarizes the characteristics of the participants.

**Table 1:** Characteristics of the study participants

Variable	Frequency (Percentage)
Age in years: median (IQR)	19.0 (17.0–22.0)
Highest education level completed	
No formal education	27 (6.5)
Some primary education	161 (38.8)
Completed primary education	131 (31.6)
Secondary education	84 (20.2)
Vocational training	9 (2.2)
Tertiary education	3 (0.7)
Current employment status	
Unemployed	162 (39.0)
Employed	8 (1.9)
Self-employed	114 (27.5)
Pupil/Student	86 (20.7)
Housewife	45 (10.8)
Marital status	
Single	209 (50.4)
Married	57 (13.7)
Cohabiting	115 (27.7)
Divorced	33 (8.0)
Widowed	1 (0.2)
Age at first marriage/union (*n* = 206)	17.0 (16.0–19.0)
Number of prior marriages/unions (*n* = 206)	
Never	10 (4.9)
Once	161 (78.2)
Twice	31 (15.0)
Three times or more	4 (1.9)
Currently living with spouse/partner. (*n* = 172)	166 (96.5)
Decision-maker for health matters	
Self	46 (11.1)
Spouse/partner	102 (24.6)
Jointly with spouse/partner	53 (12.8)
Parents	196 (47.2)
Elders	8 (1.9)
Relatives	10 (2.4)
Partner support in health matters. (*n* = 172)	
Very supportive	106 (61.6)
Somewhat supportive	60 (34.9)
Not supportive	6 (3.5)
Primary sources of income	
None	126 (30.4)
Agriculture	241 (58.1)
Business/Trade	44 (10.6)
Employment	9 (2.2)
Others	4 (0.9)
Monthly income	
No income	126 (30.4)
Less than 50,000 UGX	152 (36.6)
50,000—100,000 UGX	85 (20.5)
100,000—200,000 UGX	34 (8.2)
More than 200,000 UGX	18 (4.3)
Distance to nearest health facility	
Less than 1 km	77 (18.6)
1–5 km	242 (58.3)
6–10 km	85 (20.5)
More than 10 km	11 (2.7)
Regular means of transport to health facility	
Walking	351 (84.6)
Bicycle	18 (4.3)
Motorcycle	43 (10.4)
Motor vehicle	3 (0.7)
Ever had sexual intercourse	299 (72.0)
Age at first sexual intercourse: median (IQR) *n* = 299	16.0 (15.0–18.0)
Number of sexual partners in the past 12 months. *n* = 299	
None	20 (6.7)
One	241 (80.6)
Two	31 (10.4)
Three or more	7 (2.3)
Ever diagnosed with a sexually transmitted infection (STI)	6 (1.4)
Specify the STI diagnosis received (*n*=6)	
Gonorrhea	2 (33.3)
Syphilis	4 (66.7)
Ever been pregnant	232 (75.1)

### HIV/AIDS risk perceptions

More than two-thirds of the participants (77.8%, *n* = 323) believed they were not at risk of contracting HIV due to their current lifestyle and choices. However, 57.4% (*n* = 239) agreed that living with HIV would significantly change their life and have serious consequences. About 89.6% believed regular HIV testing and protection during sexual activities are key to maintaining health. However, only 21.0% (*n* = 117) agreed that advice from friends or family members significantly influenced their decisions about HIV testing and prevention. Table 2 provides a summary of the HIV risk perceptions among the participants.

**Table 2:** Perceptions of adolescent girls and young women in rural Northern Uganda towards HIV/AIDS

Perceptions	Frequency (Percentage)
N	415
I believe I am at risk of contracting HIV due to my current lifestyle and choices	
Strongly Disagree	194 (46.7)
Disagree	129 (31.1)
Neutral	20 (4.8)
Agree	37 (8.9)
Strongly Agree	35 (8.4)
I think that being HIV positive would significantly change my life and have serious consequences	
Strongly Disagree	50 (12.0)
Disagree	97 (23.4)
Neutral	30 (7.2)
Agree	114 (27.5)
Strongly Agree	124 (29.9)
Regular HIV testing and using protection during sexual activities are key to maintaining my health	
Strongly Disagree	9 (2.2)
Disagree	12 (2.9)
Neutral	22 (5.3)
Agree	194 (46.7)
Strongly Agree	178 (42.9)
I am confident in my ability to discuss HIV prevention with partners and insist on using protection	
Strongly Disagree	13 (3.1)
Disagree	32 (7.7)
Neutral	43 (10.4)
Agree	170 (41.0)
Strongly Agree	157 (37.8)
Advice from friends or family members significantly influences my decisions about HIV testing and prevention	
Strongly Disagree	129 (31.1)
Disagree	148 (35.7)
Neutral	21 (5.1)
Agree	80 (19.3)
Strongly Agree	37 (8.9)
Public health campaigns and messages about HIV increase my awareness and actions towards prevention	
Strongly Disagree	12 (2.9)
Disagree	22 (5.3)
Neutral	15 (3.6)
Agree	187 (45.1)
Strongly Agree	179 (43.1)
My community offers adequate support and care for individuals living with HIV/AIDS	
Strongly Disagree	125 (30.1)
Disagree	100 (24.1)
Neutral	43 (10.4)
Agree	109 (26.3)
Strongly Agree	38 (9.2)
There is a noticeable level of stigma and discrimination against HIV-positive individuals in my community	
Strongly Disagree	34 (8.2)
Disagree	111 (26.7)
Neutral	24 (5.8)
Agree	105 (25.3)
Strongly Agree	141 (34.0)
Community leaders actively participate in and promote HIV/AIDS awareness and prevention	
Strongly Disagree	137 (33.0)
Disagree	135 (32.5)
Neutral	20 (4.8)
Agree	100 (24.1)
Strongly Agree	23 (5.5)
HIV testing and treatment services are accessible to everyone in my community	
Strongly Disagree	115 (27.7)
Disagree	112 (27.0)
Neutral	18 (4.3)
Agree	90 (21.7)
Strongly Agree	80 (19.3)
Cultural beliefs in my community support safe sexual practices	
Strongly Disagree	141 (34.0)
Disagree	104 (25.1)
Neutral	86 (20.7)
Agree	74 (17.8)
Strongly Agree	10 (2.4)

### HIV testing utilization and awareness

About 75.4% (*n* = 313) had ever been tested for HIV, with 58.8% (*n* = 184) having done so within the last six months. Among those who had been tested, the majority (60.7%, *n* = 190) had their last test at a government health facility, followed by outreaches (14.7%, *n* = 46). Accessibility of HIV testing and treatment services was a concern, with over half (54.7%, *n* = 227) disagreeing that these services are accessible to everyone. Only 28.0% (*n* = 116) had heard about HIV self-testing, primarily from healthcare providers (49.1%, *n* = 57) and friends (23.3%, *n* = 27). However, only 17.5% (*n* = 25) of those aware had ever performed an HIV self-test, with most (84.0%, *n* = 21) having self-tested fewer than five times. Blood-based tests were the most common self-testing method (44.0%, *n* = 11). Table 3 summarizes HIV testing utilization among the participants.

**Table 3:** HIV testing and self-testing utilization among adolescent girls and young women in rural Northern Uganda

HIV testing (*N* = 415)	Frequency (Percentage)
Ever been tested for HIV	
Yes	313 (75.4)
No	102 (24.6)
Date of last HIV test (*n* = 313)	
Less than 6 months ago	184 (58.8)
6–12 months ago	76 (24.3)
More than a year ago	53 (16.9)
Place of last HIV test (*n* = 313)	
Government health facility	190 (60.7)
Outreaches	46 (14.7)
Medical clinic	33 (10.5)
Private hospital	23 (7.3)
Medical center	8 (2.6)
Others	13 (4.2)
Ever heard about HIV self-testing before	
Yes	116 (28.0)
No	299 (72.0)
Ever performed HIV self-testing (*n* = 116)	
No	91 (82.5)
Yes	25 (17.5)
Number of times performed HIV self-testing (*n* = 25)	
Less than 5	21 (84.0)
5 or more	4 (16.0)
HIV self-testing method used (*n* = 25)	
Blood-based test	11 (44.0)
Oral fluid/saliva-based test	8 (32.0)
Both saliva and blood-based tests	6 (24.0)
Date of last HIV self-test (*n* = 25)	
Less than six months ago	11 (44.0)
6–12 months ago	7 (28.0)
More than a year ago	7 (28.0)

### Acceptability, appropriateness, and feasibility of HIV self-testing

**Fig. 2:**
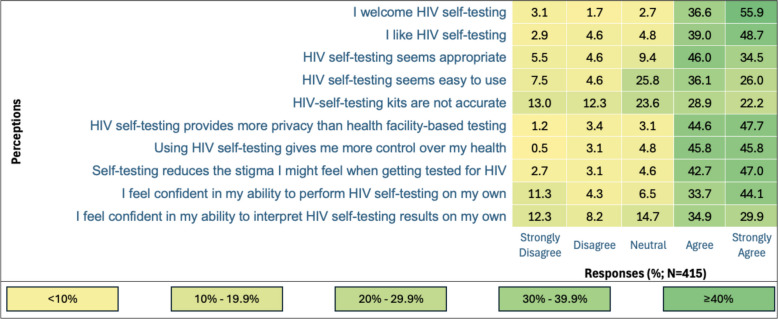
Acceptability, appropriateness, and feasibility of HIV self-testing among adolescent girls and young women in rural Northern Uganda (*N* = 415). Overall acceptability of HIVST was high, with most AGYW perceiving it as appropriate, empowering, and stigma-reducing, although concerns about accuracy remain

Most participants (92.5%, *n* = 384) welcomed HIV self-testing, and 87.7% (*n* = 364) liked the approach. Most AGYW (80.5%, *n* = 334) found it appropriate, while 62.1% (*n* = 258) believed it was easy to use. Concerns about accuracy were noted, with 51.1% (*n* = 212) believing the kits might be inaccurate, while 25.8% (*n* = 107) remained neutral. Participants valued the privacy of self-testing, with 92.3% (*n* = 383) appreciating its discretion. Similarly, 91.6% (*n* = 380) felt it provided greater control over their health, and 89.7% (*n* = 372) acknowledged its potential to reduce stigma. Confidence in performing self-testing independently was reported by 77.8% (*n* = 323), and 64.8% (*n* = 269) expressed confidence in interpreting the results (Fig. 2).

### Willingness to use HIV self-testing

**Fig. 3:**
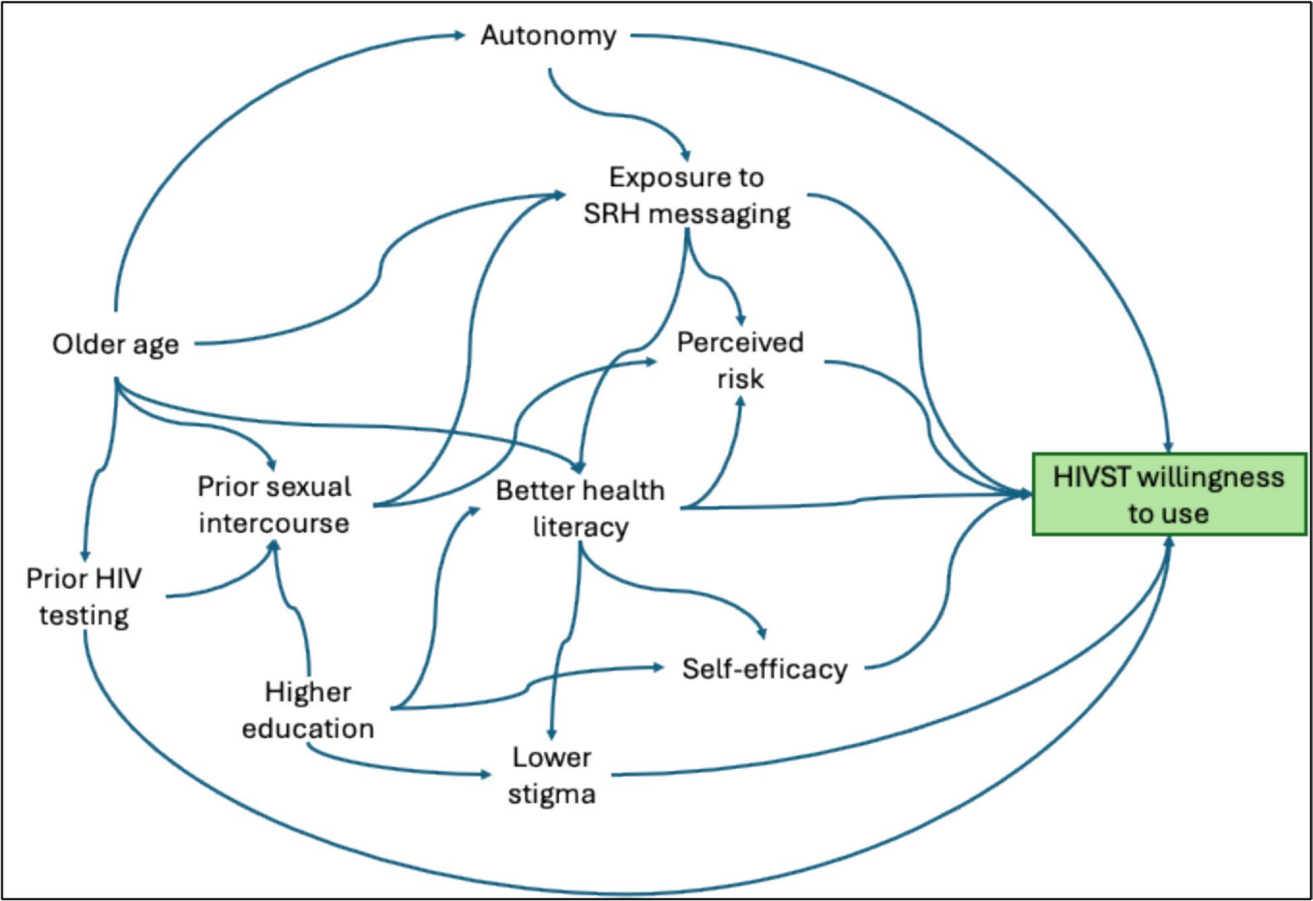
A conceptual model showing potential underlying mechanisms of the factors associated with willingness to use HIV self-tests among adolescent girls and young women in rural Northern Uganda. The model illustrates how socio-demographic factors, prior HIV testing experiences, and positive perceptions of HIVST may interact to influence willingness to adopt self-testing

Most participants (93%, *n* = 386) expressed a willingness to use HIV self-testing kits (Table 4). Willingness to use HIVST was significantly associated with age 20–24 (cOR: 2.18, 95% CI: 0.97–4.90, *p* = 0.06), completing primary education (cOR: 2.88, 95% CI: 1.28–6.50, *p* = 0.011), and ever having sexual intercourse (cOR: 2.60, 95% CI: 1.21–5.57, *p* = 0.014). Additionally, prior HIV testing experience (cOR: 2.32, 95% CI: 1.07—5.04, *p* = 0.033) and prior knowledge of HIVST (cOR: 11.88, 95% CI: 1.60–88.37, *p* = 0.016), were associated with willingness to perform HIVST. AGYW who believed HIVST offered greater privacy (cOR: 7.10, 95% CI: 2.91–17.34, *p* < 0.001), reduced stigma (cOR: 4.66, 95% CI: 1.97–11.03, *p* < 0.001), and felt confident in their ability to perform (cOR: 9.69, 95% CI: 4.24–22.17, *p* < 0.001), and interpret the results (cOR: 3.30, 95% CI: 1.51–7.19, *p* = 0.003), were more likely to be willing to perform HIVST (Table 5). Figure 3 shows the potential mechanisms through which these factors could influence the acceptance of HIVST among AGYW in rural Northern Uganda.

**Table 4:** HIV self-testing acceptability and preferences among adolescent girls and young women in rural Northern Uganda

Preferences	Frequency (Percentage)
Willingness to use HIV self-testing kits if available	
Very unwilling	14 (3.4)
Somewhat unwilling	8 (1.9)
Neutral	7 (1.7)
Willing	101 (24.3)
Very willing	285 (68.7)
Preferred HIV self-testing modality	
Blood-based test	221 (53.3)
Oral fluid/saliva-based test	192 (46.3)
None of the above	2 (0.5)
Preferred point of access of HIVST kits	
Government health facility	269 (64.8)
Community hotspots	240 (57.8)
Friends	138 (33.3)
Community health worker	91 (21.9)
Spouse/partner	91 (21.9)
Medical Clinic	81 (19.5)
Drug shop	59 (14.2)
Local council leader	15 (3.6)
Pharmacy	14 (3.4)
Need for extra support while conducting an HIV self-test	
Yes	253 (61.0)
No	162 (39.0)
Preferred source of extra support while conducting HIVST (*n* = 253)	
Healthcare workers	176 (69.6)
Friends	66 (26.1)
Partner/spouse	68 (26.9)
Parents	91 (36.0)
Others (specify):	34 (13.4)
Community leaders	6 (2.4%)
Research assistants	3 (1.2%)
Neighbours	1 (0.4%)
Community health workers	13 (5.1)
Other relatives	11 (4.3)
Comfort in disclosing HIV self-test results	
Yes	292 (70.4)
No	123 (29.6)
Person comfortable disclosing HIV self-test results to (*n*=292)	
Healthcare worker	93 (31.8)
Friends	52 (17.8)
Partner or spouse	139 (47.6)
Parents	164 (56.2)
Others (specify)	36 (12.3)
Other relatives	7 (2.4%)
Community health workers	6 (2.1%)
Not specified	1 (0.3%)
Siblings/other family members	22 (7.5)

**Table 5:** Factors associated with willingness to use HIVST kits among adolescent girls and young women in rural Northern Uganda

Variable	Willing	Not Willing	Crude OR (95% CI)	*P*-values
Age: median (IQR)	19 [17–22]	17 [15–22]	1.19 (1.03—1.37)	0.017
Age Group				
15–19	195 (50.5)	20 (69.0)	Reference	
20–24	191 (49.5)	9 (31.0)	2.18 (0.97—4.90)	0.060
Highest education level completed				
Not Completed Primary Education	168 (43.52)	20 (68.97)	Reference	
Completed Primary Education	218 (56.48)	9 (31.03)	2.88 (1.28—6.50)	0.011
Distance to nearest health facility				
Less than 5 km	302 (78.24)	17 (58.62)	Reference	
Greater than 5 km	84 (21.76)	12 (41.38)	0.39 (0.18—0.86)	0.019
Ever had sexual intercourse				
No	102 (26.4)	14 (48.3)	Reference	
Yes	284 (73.6)	15 (51.7)	2.60 (1.21—5.57)	0.014
I believe I am at risk of contracting HIV due to my current lifestyle and choices				
Disagree/Neutral	316 (81.9)	27 (93.1)	Reference	
Agree	70 (18.1)	2 (6.9)	2.99 (0.69—12.87)	0.141
My community offers adequate support and care for individuals living with HIV/AIDS				
Disagree/Neutral	311 (80.6)	20 (69.0)	Reference	
Agree	75 (19.4)	9 (31.0)	0.54 (0.23—1.22)	0.139
Ever tested for HIV?				
No	90 (23.3)	12 (41.4)	Reference	
Yes	296 (76.7)	17 (58.6)	2.32 (1.07—5.04)	0.033
Ever heard about HIV self-testing before?				
No	271 (70.2)	28 (96.6)	Reference	
Yes	115 (29.8)	1 (3.4)	11.88 (1.60—88.37)	0.016
HIV-self-testing kits are not accurate				
Disagree/Neutral	184 (47.7)	19 (65.5)	Reference	
Agree	202 (52.3)	10 (34.5)	2.09 (0.95—4.60)	0.069
HIV self-testing provides more privacy than health facility-based testing				
Disagree/Neutral	23 (6.0)	9 (31.0)	Reference	
Agree	363 (94.0)	20 (69.0)	7.10 (2.91—17.34)	< 0.001
Self-testing reduces the stigma I might feel when getting tested for HIV				
Disagree/Neutral	34 (8.8)	9 (31.0)	Reference	
Agree	352 (91.2)	20 (69.0)	4.66 (1.97—11.03)	< 0.001
I feel confident in my ability to perform HIV self-testing on my own				
Disagree/Neutral	72 (18.6)	20 (69.0)	Reference	
Agree	314 (81.4)	9 (31.0)	9.69 (4.24—22.17)	< 0.001
I feel confident in my ability to interpret HIV self-testing results on my own				
Disagree/Neutral	128 (33.2)	18 (62.1)	Reference	
Agree	258 (66.8)	11 (37.9)	3.30 (1.51—7.19)	0.003

### HIV self-testing preferences

Blood-based tests were slightly preferred (53.3%, *n* = 221) over oral fluid/saliva-based tests (46.3%, *n* = 192). The most preferred access points for self-testing kits were government health facilities (64.8%, *n* = 269) and community hotspots (57.8%, *n* = 240). Other sources included friends (33.3%, *n* = 138), community health workers (21.9%, *n* = 91), spouses/partners (21.9%, *n* = 91), and medical clinics (19.5%, *n* = 81). Extra support during self-testing was desired by 61.0% (*n* = 253), with healthcare workers being the preferred source (69.6%, *n* = 176). Most participants (70.4%, *n* = 292) were comfortable disclosing their test results, primarily to parents (56.2%, *n* = 164) and partners/spouses (47.6%, *n* = 139).

**Fig. 4:**
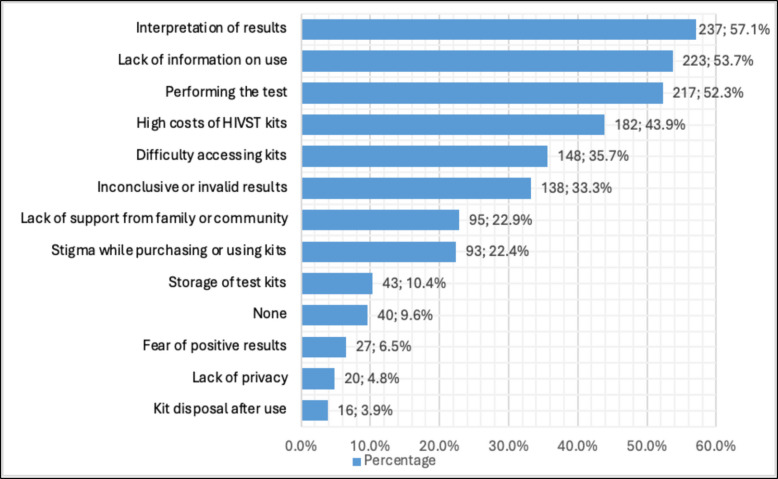
Challenges anticipated by adolescent girls and young women in rural Northern Uganda with HIV self-testing. Most concerns about HIVST clustered around usability and technical issues, including interpreting results, lack of information on use, and performing the test, whereas fewer participants raised concerns related to privacy, fear of positive results, or kit disposal

More than half of the AGYW anticipated challenges in the interpretation of the results (57.1%), lack of information on use (53.7%), and performing the tests (52.3%). Others were also concerned about the high costs of kits (43.9%) and difficulty accessing the kits (35.7%, Fig. 4).

## Discussion

This study evaluated the acceptability, appropriateness, and preferences of HIVST among AGYW in rural Northern Uganda. We observed a high level of acceptability and willingness to use HIVST, with similar preferences for both blood-based and oral fluid-based test kits. Willingness to perform HIVST was notably higher among older AGYW, those with at least primary education, prior sexual experience, and previous HIV testing.

The high acceptability and willingness to use HIVST reported in our study resonate with findings from other low-resource settings in sub-Saharan Africa, where acceptability often exceeds 80% [13]. Studies in Malawi, Zimbabwe, Zambia, the Democratic Republic of Congo, and Kenya have shown comparable enthusiasm for self-testing among young people [29–32], especially when adolescent- and youth-friendly provisions are made. Several contextual factors may explain the high acceptability observed in our study. We interpret our findings through the lens of ‘security,’ which we conceptualize as AGYW’s consideration of safety and protection from potential risks before, during, and after HIV self-testing. While not explicitly mentioned by participants, this framing helps integrate concerns around autonomy, privacy, accuracy, and access that emerged across the data.

First, prior to accepting HIVST, the autonomy that comes with it can be especially attractive in our context, where family or partners often influence healthcare decisions [13, 30, 33, 34]. This is especially applicable to our setting, where extramarital sex and infidelity are condemned among AGYW [35], and an HIV diagnosis has devastating consequences to their livelihood. Secondly, while utilizing the test, privacy and confidentiality are crucial for minimizing HIV-related stigma, and self-testing provides AGYW with a discreet means to learn their status [13, 30, 36–38]. They also consider their technical ability to perform the test accurately and whether they can trust the results, which is relevant for unfamiliar interventions like HIVST. This is influenced by their self-efficacy with HIV testing services, which is influenced either by health education or prior experience with HIV testing services. Finally, access to HIVST kits (and HIV services post-testing) presents another layer of (in)security for AGYW, given the rural nature of our study setting [39–41].

Older AGYW were more willing to use HIVST than those aged 15–19, which aligns with other studies from SSA, particularly in Kenya [42, 43], Tanzania [43], and Togo [14]. We believe older AGYW who are sexually active could have a higher perception of their risk for acquiring HIV and therefore demonstrate a higher willingness to use HIVST. This is because participants who were sexually active in our study were also more likely to accept HIVST. Additionally, older AGYW who are sexually active and have exposure to healthcare may feel more confident and willing to engage in ST because of their previous experience with sexual and health resources. Older AGYW may have also felt more secure performing the test due to better health literacy, higher self-efficacy, and greater autonomy in healthcare decisions. However, younger adolescents may face additional barriers, such as limited financial independence and stronger familial or cultural constraints regarding discussions of sexual and reproductive health. Targeted strategies, including youth-friendly health corners, school-based HIV education, and peer-led counseling by older AGYW, could help address these obstacles. Similarly, higher educational attainment was a crucial facilitator, as observed in Kenya [43], suggesting increased HIV awareness and confidence in interpreting test results. Overall, these findings highlight the necessity of tailoring interventions to ensure that younger or less experienced individuals receive targeted support.

The strong interest in HIVST among AGYW in rural Northern Uganda provides an opportunity for policymakers and program developers to expand HIV testing in this vulnerable population. Practical measures could include integrating HIVST into routine antenatal and postnatal care [44–46] or outpatient settings [17], establishing youth-friendly pop-up clinics at community events [47, 48], and scaling up school-based campaigns [49]. Efforts should be made to promote HIVST among healthcare workers at health facilities that AGYW suggested as favored access points, especially because this has been the main source of HIVST in Omoro district. Enlisting community health workers or peer educators to distribute free or subsidized kits and offer real-time guidance could also extend reach in remote areas. All these efforts should be accompanied by active awareness campaigns through preferred information sources to generate demand among AGYW. In parallel, programs should ensure clear referral pathways for confirmatory testing and linkage to care [50], potentially through mobile clinics or digital platforms, so that positive results lead promptly to professional support. Such integrated, multi-pronged efforts can protect privacy, address accuracy concerns, and normalize HIVST, thereby accelerating progress toward the UNAIDS 95–95-95 targets in settings with historically low testing uptake.

Despite its high acceptability, several factors may limit HIVST uptake among AGYW in rural Northern Uganda. Over half of the participants in our study expressed concerns about the accuracy of the kits. This hesitancy may be rooted in limited exposure to reliable information about HIVST, pointing to the need for comprehensive counseling, clear user instructions, and robust community sensitization campaigns to increase confidence. This could particularly explain why slightly more participants preferred blood-based rather than oral-based HIVST, as seen among men in a previous systematic review [51]. Cultural norms and family dynamics can also discourage younger adolescents from independently seeking HIVST, emphasizing the need for discreet access points and supportive counseling [52, 53]. Furthermore, long distances, high travel costs, and logistical challenges in remote areas could present significant barriers [13], as many rural health facilities lack the resources to stock subsidized kits, risking unequal access. In closely-knit communities like rural Northern Uganda, the stigma surrounding HIV, fueled by fears of judgment and social isolation, further complicates these issues. We must address these challenges if HIVST is to reach all AGYW who may need it. Implementation research will be crucial in identifying how these bottlenecks can be addressed in real-world settings.

## Strengths and limitations

A key strength of this study is its community-based design and 100% response rate, which minimized selection bias and strengthened the representativeness of the sample. These findings offer valuable insights into a hardly reached population crucial for HIV prevention efforts. However, certain limitations warrant consideration while interpreting the findings. First, the adapted version of AIM, IAM, and FIM was not validated in our setting. Secondly, self-reported data may introduce recall and social desirability biases, which could lead participants to overstate their acceptance of HIVST or underreport socially undesirable behaviors. Finally, the cross-sectional nature of the research restricts causal inferences about factors such as prior testing experience and willingness to self-test. Therefore, while this study strengthens the evidence base on HIVST among AGYW, caution is advised when generalizing these results to other regions or contexts.

## Conclusion

HIV self-testing is highly acceptable among AGYW in rural Northern Uganda, largely because of its privacy, autonomy, and convenience. To realize its full potential in increasing HIV testing rates, diagnosis, and linkage to care, health policies must prioritize broad access to self-testing kits through government facilities, community-based outlets, or integrated programs to reach younger adolescents, individuals with limited education, and those who have never tested. Equally important is providing clear instructions, supportive counseling, and efficient linkage to care for those who test positive. These would address major concerns raised by participants, particularly around the accuracy of HIVST kits, stigma, and access to reliable and convenient testing options. Future research on longitudinal uptake, correct usage, and post-test follow-up will be essential for refining implementation strategies to improve early HIV diagnosis, streamline linkage to treatment, and ultimately reduce HIV incidence.

## Supplementary Information

Supplementary Material 1.

## Data Availability

All data and materials from this study will be available upon reasonable request from the corresponding author (Dr. Ronald Olum) and relevant institutional approval.

## Abbreviations

AGYW: Adolescent girls and young women
ART: Antiretroviral therapy
CHWs: Community health workers
FGD: Focus group discussion
HBM: Health-belief model
HIVST: HIV self-testing
HTS: HIV testing services
KII: Key-informant interviews
SSA: Sub-Saharan Africa
UNAIDS: Joint United Nations Programme on HIV/AIDS

## References

[1] Joint United Nations Programme on HIV/AIDS. The urgency of now: AIDS at a crossroads. Geneva: 2024. https://www.unaids.org/en/resources/documents/2024/global-aids-update-2024.

[2] Joint United Nations Programme on HIV/AIDS. AIDSinfo: Global data on HIV epidemiology and response. https://aidsinfo.unaids.org/. Accessed 12 Jan 2025.

[3] Leung Soo C, Pant Pai N, Bartlett SJ, Esmail A, Dheda K, Bhatnagar S. Socioeconomic factors impact the risk of HIV acquisition in the township population of South Africa: a Bayesian analysis. PLOS Glob Public Health. 2023;3(1):e0001502. 36963084 10.1371/journal.pgph.0001502 PMC10021863

[4] Joint United Nations Programme on HIV/AIDS: The path that ends AIDS: UNAIDS Global AIDS Update 2023. In*.* Geneva, Switzerland: UNAIDS; 2023. https://www.unaids.org/en/resources/documents/2023/global-aids-update-2023.

[5] Kumah E, Boakye DS, Boateng R, Agyei E. Advancing the global fight against HIV/Aids: strategies, barriers, and the road to eradication. Ann Glob Health. 2023;89(1):83. 38046536 10.5334/aogh.4277 PMC10691281

[6] Giguere K, Eaton JW, Marsh K, Johnson LF, Johnson CC, Ehui E, et al. Trends in knowledge of HIV status and efficiency of HIV testing services in sub-Saharan Africa, 2000–20: a modelling study using survey and HIV testing programme data. Lancet HIV. 2021;8(5):e284–93. 33667411 10.1016/S2352-3018(20)30315-5 PMC8097636

[7] Zhang Y, Johnson CC, Nguyen VTT, Ong JJ. Role of HIV self-testing in strengthening HIV prevention services. Lancet HIV. 2024;11(11):e774–82. 39332440 10.1016/S2352-3018(24)00187-5

[8] Obiezu-Umeh C, Gbajabiamila T, Ezechi O, Nwaozuru U, Ong JJ, Idigbe I, et al. Young people’s preferences for HIV self-testing services in Nigeria: a qualitative analysis. BMC Public Health. 2021;21(1):67. 33413246 10.1186/s12889-020-10072-1 PMC7792110

[9] O’Reilly A, Mavhu W, Neuman M, Kumwenda MK, Johnson CC, Sinjani G, et al. Accuracy of and preferences for blood-based versus oral-fluid-based HIV self-testing in Malawi: a cross-sectional study. BMC Infect Dis. 2024;22(Suppl 1):979. 38566003 10.1186/s12879-024-09231-1 PMC10985843

[10] Inwani I, Chhun N, Agot K, Cleland CM, Rao SO, Nduati R, et al. Preferred HIV testing modalities among adolescent girls and young women in Kenya. J Adolesc Health. 2021;68(3):497–507. 32792256 10.1016/j.jadohealth.2020.07.007

[11] Ekouevi DK, Bitty-Anderson AM, Gbeasor-Komlanvi FA, Coffie AP, Eholie SP. HIV self-testing: the key to unlock the first 90 in West and Central Africa. Int J Infect Dis. 2020;95:162–6. 32070722 10.1016/j.ijid.2020.02.016

[12] Qin Y, Tang W, Nowacki A, Mollan K, Reifeis SA, Hudgens MG, et al. Benefits and potential harms of human immunodeficiency virus self-testing among men who have sex with men in China: an implementation perspective. Sex Transm Dis. 2017;44(4):233–8. 28282650 10.1097/OLQ.0000000000000581 PMC5347468

[13] Zeleke EA, Stephens JH, Gesesew HA, Gello BM, Ziersch A. Acceptability and use of HIV self-testing among young people in sub-Saharan Africa: a mixed methods systematic review. BMC Prim Care. 2024;25(1):369. 39407123 10.1186/s12875-024-02612-0 PMC11475945

[14] Sadio AJ, Kouanfack HR, Konu RY, Gbeasor-Komlanvi FA, Azialey GK, Gounon HK, et al. HIV self-testing: a highly acceptable and feasible strategy for reconnecting street adolescents with HIV screening and prevention services in Togo (the STADOS study). PLoS ONE. 2024;19(10):e0312693. 39446800 10.1371/journal.pone.0312693 PMC11500916

[15] Pettifor A, Lippman SA, Kimaru L, Haber N, Mayakayaka Z, Selin A, et al. HIV self-testing among young women in rural South Africa: A randomized controlled trial comparing clinic-based HIV testing to the choice of either clinic testing or HIV self-testing with secondary distribution to peers and partners. EClinicalMedicine. 2020;21:100327. 10.1016/j.eclinm.2020.100327. 10.1016/j.eclinm.2020.100327 PMC7171186 32322811

[16] Nakalega R, Mukiza N, Menge R, Kizito S, Babirye JA, Kuteesa CN, et al. Feasibility and acceptability of peer-delivered HIV self-testing and PrEP for young women in Kampala, Uganda. BMC Public Health. 2023;23(1):1163. 37322510 10.1186/s12889-023-16081-0 PMC10273744

[17] Dovel K, Shaba F, Offorjebe OA, Balakasi K, Nyirenda M, Phiri K, et al. Effect of facility-based HIV self-testing on uptake of testing among outpatients in Malawi: a cluster-randomised trial. Lancet Glob Health. 2020;8(2):e276–87. 31981557 10.1016/S2214-109X(19)30534-0

[18] Terefe B, Jembere MM, Reda GB, Asgedom DK, Assefa SK, Lakew AM. Knowledge, and utilization of HIV self-testing, and its associated factors among women in sub-Saharan Africa: evidence from 21 countries demographic and health survey. BMC Public Health. 2024;24(1):1960. 39044258 10.1186/s12889-024-19529-z PMC11265320

[19] Segawa I, Bakeera-Kitaka S, Ssebambulidde K, Muwonge TR, Oriokot L, Ojiambo KO, et al. Factors associated with HIV self-testing among female university students in Uganda: a cross-sectional study. AIDS Res Ther. 2022;19(1):59. 36457098 10.1186/s12981-022-00484-x PMC9713199

[20] Matovu JKB, Nambuusi A, Nakabirye S, Wanyenze RK, Serwadda D. Formative research to inform the development of a peer-led HIV self-testing intervention to improve HIV testing uptake and linkage to HIV care among adolescents, young people and adult men in Kasensero fishing community, Rakai, Uganda: a qualitative study. BMC Public Health. 2020;20(1):1582. 33081735 10.1186/s12889-020-09714-1 PMC7576713

[21] Matovu JKB, Bogart LM, Nakabugo J, Kagaayi J, Serwadda D, Wanyenze RK, et al. Feasibility and acceptability of a pilot, peer-led HIV self-testing intervention in a hyperendemic fishing community in rural Uganda. PLoS ONE. 2020;15(8):e0236141. 32764751 10.1371/journal.pone.0236141 PMC7413506

[22] Matovu JKB, Nambuusi A, Wanyenze RK, Serwadda D. Peer-leaders’ experiences and challenges in distributing HIV self-test kits in a rural fishing community, Rakai, Uganda. BMC Public Health. 2021;21(1):708. 33845811 10.1186/s12889-021-10804-x PMC8042983

[23] Muyinda H, Jongbloed K, Zamar DS, Malamba SS, Ogwang MD, Katamba A, et al. Cango lyec (healing the elephant): HIV prevalence and vulnerabilities among adolescent girls and young women in postconflict northern Uganda. J Acquir Immune Defic Syndr. 2023;94(2):95–106. 37276188 10.1097/QAI.0000000000003234 PMC10497204

[24] Olum R, Geng EH, Kitutu FE, Musoke PM. Feasibility, acceptability and preliminary effect of a community-led HIV self-testing model among adolescent girls and young women in rural northern Uganda: a quasi-experimental study protocol. Implement Sci Commun. 2024;5(1):56. 38773505 10.1186/s43058-024-00596-7 PMC11110295

[25] Wang X, Rafa M, Moyer JD, Li J, Scheer J, Sutton P. Estimation and mapping of sub-national GDP in Uganda using NPP-VIIRS imagery. Remote Sens. 2019;11(2):163.

[26] Uganda Bureau of Statistics: The National Population and Housing Census 2014 – Area Specific Profile Series (Omoro District). In*.* Edited by UBOS. Kampala, Uganda; 2017. https://www.ubos.org/wpcontent/uploads/publications/2014CensusProfiles/OMORO.pdf.

[27] Proctor E, Silmere H, Raghavan R, Hovmand P, Aarons G, Bunger A, et al. Outcomes for implementation research: conceptual distinctions, measurement challenges, and research agenda. Adm Policy Ment Health. 2011;38(2):65–76. 20957426 10.1007/s10488-010-0319-7 PMC3068522

[28] Weiner BJ, Lewis CC, Stanick C, Powell BJ, Dorsey CN, Clary AS, et al. Psychometric assessment of three newly developed implementation outcome measures. Implement Sci. 2017;12(1):108. 28851459 10.1186/s13012-017-0635-3 PMC5576104

[29] Hatzold K, Gudukeya S, Mutseta MN, Chilongosi R, Nalubamba M, Nkhoma C, et al. HIV self-testing: breaking the barriers to uptake of testing among men and adolescents in sub-Saharan Africa, experiences from STAR demonstration projects in Malawi, Zambia and Zimbabwe. J Int AIDS Soc. 2019;22(Suppl 1):e25244. 30907505 10.1002/jia2.25244 PMC6432104

[30] Indravudh PP, Sibanda EL, d’Elbee M, Kumwenda MK, Ringwald B, Maringwa G, et al. “I will choose when to test, where I want to test”: investigating young people’s preferences for HIV self-testing in Malawi and Zimbabwe. AIDS (London, England). 2017;31(Suppl 3):S203–12. 28665878 10.1097/QAD.0000000000001516 PMC5497773

[31] Wilson KS, Mugo C, Katz DA, Manyeki V, Mungwala C, Otiso L, et al. High acceptance and completion of HIV self-testing among diverse populations of young people in Kenya using a community-based distribution strategy. AIDS Behav. 2022;26(3):964–74. 34468968 10.1007/s10461-021-03451-1 PMC8409270

[32] Izizag BB, Situakibanza H, Mbutiwi T, Ingwe R, Kiazayawoko F, Nkodila A, et al. Factors associated with acceptability of HIV self-testing (HIVST) among university students in a peri-urban area of the Democratic Republic of Congo (DRC). Pan Afr Med J. 2018;31:248. 31452830 10.11604/pamj.2018.31.248.13855 PMC6693788

[33] Lapsley R, Beima-Sofie K, Moraa H, Manyeki V, Mung’ala C, Kohler PK, et al. “They have given you the morale and confidence:” adolescents and young adults want more community-based oral HIV self-testing options in Kenya. AIDS Care. 2023;35(3):392–8. 35468010 10.1080/09540121.2022.2067315 PMC9592677

[34] Hermanus T, O’Grady M. A principle-based approach to justify the use of HIV self-testing in South Africa. Dev World Bioeth. 2022;22(1):53–62. 34075703 10.1111/dewb.12319

[35] Patel SH, Muyinda H, Sewankambo NK, Oyat G, Atim S, Spittal PM. In the face of war: examining sexual vulnerabilities of Acholi adolescent girls living in displacement camps in conflict-affected Northern Uganda. BMC Int Health Hum Rights. 2012;12:38. 23270488 10.1186/1472-698X-12-38 PMC3536565

[36] Koris AL, Stewart KA, Ritchwood TD, Mususa D, Ncube G, Ferrand RA, et al. Youth-friendly HIV self-testing: acceptability of campus-based oral HIV self-testing among young adult students in Zimbabwe. PLoS ONE. 2021;16(6):e0253745. 34185815 10.1371/journal.pone.0253745 PMC8241036

[37] Kumwenda M, Munthali A, Phiri M, Mwale D, Gutteberg T, MacPherson E, et al. Factors shaping initial decision-making to self-test amongst cohabiting couples in urban Blantyre, Malawi. AIDS Behav. 2014;18(Suppl 4):S396-404. 24929834 10.1007/s10461-014-0817-9 PMC4102820

[38] Bwalya C, Simwinga M, Hensen B, Gwanu L, Hang’andu A, Mulubwa C, et al. Social response to the delivery of HIV self-testing in households: experiences from four Zambian HPTN 071 (PopART) urban communities. AIDS Res Ther. 2020;17(1):32. 32527261 10.1186/s12981-020-00287-y PMC7288417

[39] Chikwari CD, Dringus S, Ferrand RA. Barriers to, and emerging strategies for, HIV testing among adolescents in sub-Saharan Africa. Curr Opin HIV AIDS. 2018;13(3):257–64. 29401121 10.1097/COH.0000000000000452

[40] Strauss M, Rhodes B, George G. A qualitative analysis of the barriers and facilitators of HIV counselling and testing perceived by adolescents in South Africa. BMC Health Serv Res. 2015;15:250. 26123133 10.1186/s12913-015-0922-0 PMC4484707

[41] Ssali PW, Kintu TM, Karungi I, Namuyaba AK, Kyagambiddwa T, Namaseruka R, et al. “If you find that I am HIV positive, don’t tell me”: Exploring the barriers and recommendations for HIV prevention services utilization among youth in rural southwestern Uganda. PLOS Glob Public Health. 2024;4(9):e0002555. 39269977 10.1371/journal.pgph.0002555 PMC11398690

[42] Inwani I, Chhun N, Agot K, Cleland CM, Rao SO, Nduati R, et al. Preferred HIV testing modalities among adolescent girls and young women in Kenya. The Journal of adolescent health : official publication of the Society for Adolescent Medicine. 2021;68(3):497–507. 32792256 10.1016/j.jadohealth.2020.07.007

[43] Olakunde BO, Alemu D, Conserve DF, Mathai M, Mak’anyengo MO, Jennings Mayo-Wilson L. Awareness of and willingness to use oral HIV self-test kits among Kenyan young adults living in informal urban settlements: a cross-sectional survey. AIDS Care. 2023;35(9):1259–69. 35266433 10.1080/09540121.2022.2050176 PMC9463408

[44] Zishiri V, Conserve DF, Haile ZT, Corbett E, Hatzold K, Meyer-Rath G, et al. Secondary distribution of HIV self-test kits by HIV index and antenatal care clients: implementation and costing results from the STAR Initiative in South Africa. BMC Infect Dis. 2023;22(Suppl 1):971. 37264343 10.1186/s12879-023-08324-7 PMC10234581

[45] Thirumurthy H, Masters SH, Mavedzenge SN, Maman S, Omanga E, Agot K. Promoting male partner HIV testing and safer sexual decision making through secondary distribution of self-tests by HIV-negative female sex workers and women receiving antenatal and post-partum care in Kenya: a cohort study. Lancet HIV. 2016;3(6):e266-274. 27240789 10.1016/S2352-3018(16)00041-2 PMC5488644

[46] Bouba Y, Djomo ARD, Mouliom FN, Souleymanou A, Lifanda E, Liman Y, et al. Evaluating the effectiveness of oral HIV self testing according to distribution models in Cameroon. Sci Rep. 2024;14(1):30694. 39730382 10.1038/s41598-024-78444-w PMC11681156

[47] Nwaozuru U, Iwelunmor J, Ong JJ, Salah S, Obiezu-Umeh C, Ezechi O, et al. Preferences for HIV testing services among young people in Nigeria. BMC Health Serv Res. 2019;19(1):1003. 31881959 10.1186/s12913-019-4847-x PMC6935128

[48] Nwaozuru U, Tahlil KM, Obiezu-Umeh C, Gbaja-Biamila T, Asuquo SE, Idigbe I, et al. Tailoring youth-friendly health services in Nigeria: a mixed-methods analysis of a designathon approach. Glob Health Action. 2021;14(1):1985761. 34904539 10.1080/16549716.2021.1985761 PMC8676684

[49] Mukora-Mutseyekwa F, Mundagowa PT, Kangwende RA, Murapa T, Tirivavi M, Mukuwapasi W, et al. Implementation of a campus-based and peer-delivered HIV self-testing intervention to improve the uptake of HIV testing services among university students in Zimbabwe: the SAYS initiative. BMC Health Serv Res. 2022;22(1):222. 35177055 10.1186/s12913-022-07622-1 PMC8855554

[50] Hlongwa M, Moyo E, Dzinamarira T. Approaches for improving linkage to HIV care among HIV self-testing individuals in sub-Saharan Africa. BMJ Glob Health. 2023;8(7):e012664. 10.1136/bmjgh-2023-012664. 10.1136/bmjgh-2023-012664 PMC10351227 37451688

[51] Adepoju VA, Imoyera W, Onoja AJ. Preferences for oral- vs blood-based human immunodeficiency virus self-testing: a scoping review of the literature. World J Methodol. 2023;13(3):142–52. 37456972 10.5662/wjm.v13.i3.142 PMC10348079

[52] Mason S, Ezechi OC, Obiezu-Umeh C, Nwaozuru U, BeLue R, Airhihenbuwa C, et al. Understanding factors that promote uptake of HIV self-testing among young people in Nigeria: framing youth narratives using the PEN-3 cultural model. PLoS ONE. 2022;17(6):e0268945. 35657809 10.1371/journal.pone.0268945 PMC9165856

[53] Catania JA, Huun C, Dolcini MM, Urban AJ, Fleury N, Ndyetabula C, et al. Overcoming cultural barriers to implementing oral HIV self-testing with high fidelity among Tanzanian youth. Transl Behav Med. 2021;11(1):87–95. 31785201 10.1093/tbm/ibz157 PMC8344299

